# Impact of Nutrient Type and Sequence on Glucose Tolerance: Physiological Insights and Therapeutic Implications

**DOI:** 10.3389/fendo.2019.00144

**Published:** 2019-03-08

**Authors:** Lorenzo Nesti, Alessandro Mengozzi, Domenico Tricò

**Affiliations:** ^1^Department of Clinical and Experimental Medicine, University of Pisa, Pisa, Italy; ^2^Sant'Anna School of Advanced Studies, Institute of Life Sciences, Pisa, Italy

**Keywords:** macronutrient preloads, food order, gastric emptying, glucose tolerance, insulin secretion, postprandial glycemia, medical nutrition therapy, type 2 diabetes

## Abstract

Pharmacological and dietary interventions targeting postprandial glycemia have proved effective in reducing the risk for type 2 diabetes and its cardiovascular complications. Besides meal composition and size, the timing of macronutrient consumption during a meal has been recently recognized as a key regulator of postprandial glycemia. Emerging evidence suggests that premeal consumption of non-carbohydrate macronutrients (i.e., protein and fat “preloads”) can markedly reduce postprandial glycemia by delaying gastric emptying, enhancing glucose-stimulated insulin release, and decreasing insulin clearance. The same improvement in glucose tolerance is achievable by optimal timing of carbohydrate ingestion during a meal (i.e., carbohydrate-last meal patterns), which minimizes the risk of body weight gain when compared with nutrient preloads. The magnitude of the glucose-lowering effect of preload-based nutritional strategies is greater in type 2 diabetes than healthy subjects, being comparable and additive to current glucose-lowering drugs, and appears sustained over time. This dietary approach has also shown promising results in pathological conditions characterized by postprandial hyperglycemia in which available pharmacological options are limited or not cost-effective, such as type 1 diabetes, gestational diabetes, and impaired glucose tolerance. Therefore, preload-based nutritional strategies, either alone or in combination with pharmacological treatments, may offer a simple, effective, safe, and inexpensive tool for the prevention and management of postprandial hyperglycemia. Here, we survey these novel physiological insights and their therapeutic implications for patients with diabetes mellitus and altered glucose tolerance.

## Conceptual Framework

Type 2 diabetes (T2D) affects more than 400 million people worldwide and its prevalence is constantly increasing (1). The first metabolic alteration detectable in the progression of the disease is typically a loss of postprandial glucose control (2, 3), which is an independent risk factor for T2D (4, 5) and its complications (5–11). Targeting postprandial glycemia has proved effective for reducing the incidence of T2D (12–14). However, pharmacological control of postprandial glucose in the prediabetic stage rises ethical and economic concerns, and novel cost-effective treatments are required.

Medical nutrition therapy is recommended as first line treatment for prediabetes and T2D (15, 16) and may be a useful tool for improving glucose tolerance. In fact, meal composition and size have a profound impact on the physiological processes that regulate postprandial glycemia, such as gastric emptying and intestinal glucose absorption, pancreatic, and gut hormone release, hepatic insulin extraction, glucose uptake by insulin-sensitive tissues, and endogenous glucose production (17). Adherence to lifelong nutritional interventions involving energy restriction is often poor, and therefore alternative dietary strategies focusing on eating patterns are gaining growing interest.

**Figure 1:**
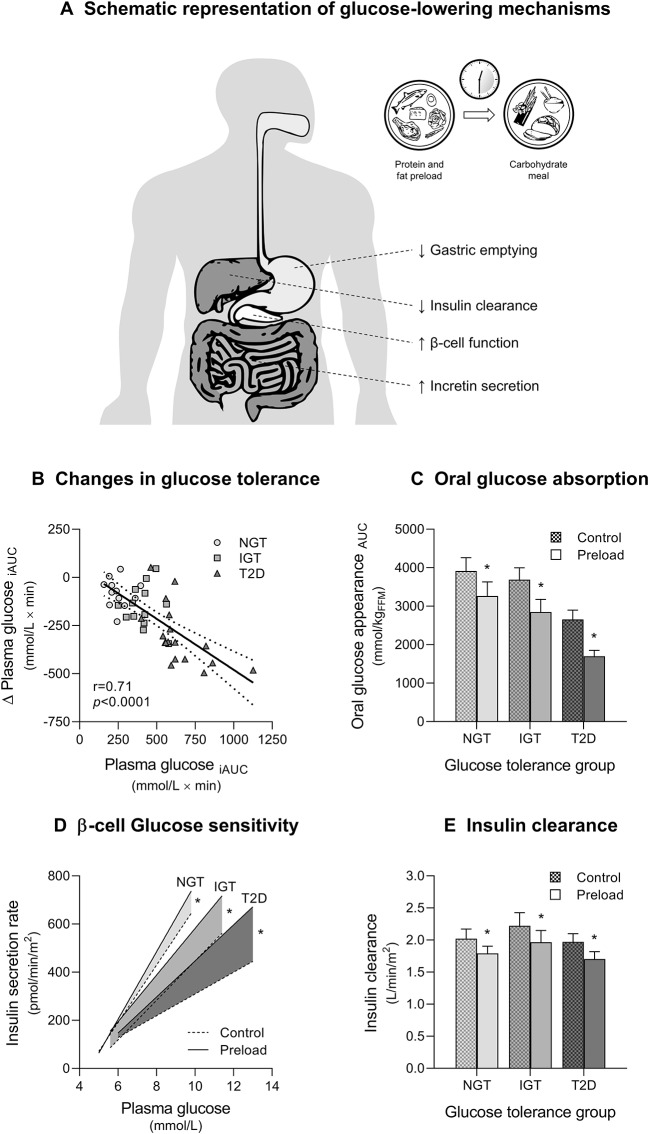
Glucose-lowering effects of mixed nutrient preloads. **(A)** Schematic representation of postprandial glucose-lowering mechanisms activated by nutrient preloads. **(B)** Reduction of postload glucose excursions (Δ Plasma glucose _iAUC_) during a 75 g oral glucose tolerance test (OGTT) after a mixed nutrient preload is proportional to the degree of glucose tolerance (Plasma glucose _iAUC_) in subjects with normal glucose tolerance (NGT), impaired glucose tolerance (IGT), and type 2 diabetes (T2D). The physiological mechanisms responsible for the improvement in glucose tolerance during a 75 g OGTT preceded by a mixed nutrient preload (Preload) compared with a control OGTT (Control) are: **(C)** decreased rate of appearance of oral glucose for delayed gastric emptying; **(D)** enhanced glucose-stimulated insulin secretion (β-cell glucose sensitivity); and **(E)** reduced insulin clearance. Data are pooled from Trico et al. (18) and Trico et al. (19), for a total of 43 subjects examined (12 NGT, 13 IGT, and 18 T2D, except for B where T2D = 10). ^*^p < 0.05 using paired Wilcoxon signed-rank test for within-group difference between Preload and Control.

One emerging approach is premeal ingestion of non-carbohydrate macronutrients (namely protein and fat), which has been shown to reduce postprandial hyperglycemia in both T2D and at-risk individuals (Figure 1A) (20). It has long been known that non-carbohydrate components of the meal can markedly influence postprandial glycemia (18, 21–25). More recently, the magnitude of the glucose-lowering effect of protein and fat was found to be even greater when these macronutrients are consumed before carbohydrates than mixed with them (22, 23). Based on these observations, “preloading” each meal with protein and fat or tailoring the sequence of macronutrient ingestion (i.e., consuming protein- and fat-rich food before carbohydrate) has been proposed as a novel strategy for the prevention and management of postprandial hyperglycemia.

The number of experimental studies in support to the clinical application of this promising nutritional approach is rapidly growing. However, gathering all available evidence is challenging given the different keywords used by different groups to define similar dietary strategies [e.g., protein/fat/nutrient premeal consumption (26, 27) or preload (19, 23, 28–30), food/meal/nutrient sequence (31, 32) or order (33)]. A further degree of complexity in the interpretation and comparison of different findings is produced by the heterogeneity of study designs. In fact, the effect of preload-based nutritional interventions on postprandial glycemia appears largely dependent upon different variables, such as preload composition, size, and timing of ingestion, test meal stimulus, and individual glucose tolerance status (20) (Figure 1B).

Herein, we review the available evidence on the acute and chronic effect of protein and fat preloads on postprandial glycemia throughout the whole spectrum of glucose tolerance, from diabetic patients to prediabetic and healthy individuals (Table 1), the underpinning physiological mechanisms, and the potential therapeutic relevance in different clinical settings.

**Table 1:** Available studies examining the glucose-lowering effects of preload-based nutritional interventions.

**References**	***n***	**Preload**	**Timing**	**Control**	**Test meal**	**Effect on postload glycemia**
**TYPE 2 DIABETES**						
Gentilcore et al. (22)	6	30 ml olive oil	−30′	30 ml water	65 g powdered potato + 20 g glucose + 250 ml water	Delayed glucose peak
Ma et al. (23)	8	55 g whey protein + 350 ml water	−30′	350 ml water	65 g powdered potato + 20 g glucose + 250 ml water	Glucose iAUC −51%
Chen et al. (34)	10	30 g soya beans + 75 g yogurt	−120′	None	51 g carbohydrate + 4.8 g fat +5.8 g protein	Glucose iAUC −36% 2 h glucose −9%
Clifton et al. (35)	24	17 g whey protein + 3 g lactose + 5 g guar + 150 ml water	−15′	150 ml water	2–3 slices of bread + jam and margarine, tea/coffee	Peak glucose −1.4 mM Mean glucose −0.8 mM
Jakubovicz et al. (36)	15	50 g whey protein + 250 ml water	−30′	250 ml water	High-glycemic index breakfast (353 kcal)	Glucose AUC −28%
Li et al. (28)	30	18 g Inzone ® Vitality (7.6 g protein + 1.8 g fat + 1.6 g fiber + 5.2 g carbs) + 150 ml water	−30′, each meal,12 weeks	None	Normal diet	HbA1c −0.3% 2 h glucose −14%
Ma et al. (37)	7	25 g whey protein + 100 ml water	−30′, 4 weeks	100 ml flavored water	Normal diet *Acute studies*: 65 g potato + 20 g glucose + 250 ml water	Fructosamine −9%^*^ Peak glucose −5^*^-9%
Shukla et al. (33)	11	150 g chicken meat + 170 g vegetables	−15′	Reverse order	90 g ciabatta bread + 120 ml orange juice	Glucose iAUC −73% 2 h glucose −7%
Trico et al. (18)	10	50 g parmesan cheese + 50 g egg + 300 ml water	−30′	500 ml water	75 g oral glucose	Glucose iAUC−49%
Kuwata et al. (31)	12	100 g mackerel fish or 79 g beef meat	−15′	Reverse order	150 g rice	Glucose iAUC −30 to 40%
Trico et al. (19)	8	50 g parmesan cheese + 50 g egg + 300 ml water	−30′	500 ml water	75 g oral glucose	Glucose iAUC −28% Peak glucose −49%
Trico et al. (38)	17	Protein- and fat-rich food before carbohydrate	Before 2 meals, 8 weeks	Reverse order	Isocaloric diet	HbA1c −0.3% ^*^ Glucose CV −32% 2 h glucose rise −102%
Wu et al. (30)	22	25 g whey protein + 250 ml water	−30′	250 ml flavored water	400 g beef lasagna	Glucose AUC −1% ^*^ Peak glucose−5%
Shukla et al. (39)	16	150 g chicken meat + 170 g vegetables	−10′	Reverse order	90 g ciabatta bread + 120 ml orange juice	Glucose iAUC −53% Peak glucose −54%
Bae et al. (27)	15	30 g protein- and fiber-rich bar + 150 ml water	−30′	Reverse order	100 g bagel + 70 g cheese + 210 ml orange juice	Glucose iAUC−25%
Watson et al. (40)	79	17 g whey protein + 5 g guar gum + 150 ml water	15′ before 2 meals, 12 weeks	150 ml flavored water	65 g powdered potato + 1 egg yolk + 20 g glucose + 200 ml water	HbA1c −0.1% Peak glucose −15%
Watson et al. (41)	21	17 g whey protein ± 5 g guar gum + 60 mg sucralose + 150 ml water	−15′	60 mg sucralose + 150 ml water	65 g powdered potato + 1 egg yolk + 20 g glucose + 200 ml water	Glucose iAUC −15% (independent of guar gum consumption)
**IMPAIRED GLUCOSE TOLERANCE**						
Trico et al. (18)	12	50 g parmesan cheese + 50 g egg + 300 ml water	−30′	500 ml water	75 g oral glucose	Glucose iAUC −37%
Crouch and Slater (42)	20	14.2 g almonds + 237 ml water	−30'	None	75 g oral glucose	Glucose AUC −16% 2 h glucose −14%
Shukla et al. (43)	15	100 g chicken meat + 285 vegetables + 15 ml olive oil	−20′	Reverse order	90 g ciabatta bread	Glucose iAUC −39%
**NORMAL GLUCOSE TOLERANCE**						
Cunningham and Read (21)	6	60 g margarine	−20′	300 ml beef consommé	300 g mashed potato + 230 ml water	Glucose AUC −39% Peak glucose −18% Delayed glucose peak
Akhavan et al. (26)	16	5–40 g whey protein + 400 ml water	−30′	300 ml water	12 kcal/kg pizza + 500 ml water	Dose-dependent reduction in glucose AUC (~0–50%)
Akhavan et al. (44)	10	10 or 20 g whey protein + 400 ml water	−30′	300 ml water	12 kcal/kg pizza + 500 ml water	Mean glucose −4%
Trico et al. (18)	12	50 g parmesan cheese + 50 g egg + 300 ml water	−30'	500 ml water	75 g oral glucose	Glucose iAUC −32%
Kuwata et al. (31)	10	100 g mackerel fish or 79 g beef meat	15′	Reverse order	150 g rice	Glucose AUC −19 to 30% ^*^
Sun et al. (45)	20	322 ml soy or dairy milk	−30′	None	91 g white bread + 322 ml water	Glucose iAUC −40 to 49%
Nishino et al. (46)	8	60 g pork meat + 150 g vegetables + 5 ml olive oil	Before carbs	Reverse order	150 g rice + 45 g pumpkin + 75 g orange + 150 ml water	Glucose AUC −48% ^*^
Bae et al. (27)	15	30 g protein- and fiber-rich bar + 150 ml water	−30′	Reverse order	100 g bagel + 70 g cheese + 210 ml orange juice	Glucose iAUC −18%
**TYPE 1 DIABETES**						
Faber et al. (47)	20	22 g cheese + 30 g turkey meat	−15′	22 g cheese + 30 g turkey meat in test meal	2 slices bread + 15 g jam + 150 ml orange juice	Glucose AUC −19% ^*^ Mean glucose −9%
**GESTATIONAL DIABETES**						
Li et al. (29)	33 each group	18 g Inzone ^®;^ Vitality (7.6 g protein + 1.8 g fat + 1.6 g fiber + 5.2 g carbs) + 250 ml water	−30′, each meal, 7 weeks	18 g milk powder (3.5 protein + 1.1 g fat + 11.2 g carbs) + 250 ml water	Normal diet	Fasting glucose ~-17%2 h glucose ~-5%

*P < 0.05 unless otherwise specified*.

* *P = ns. AUC, area under the curve; CV, coefficient of variation; iAUC, incremental AUC*.

## Impact of Macronutrient Preloads on Postprandial Glycemia

### Type 2 Diabetes

In subjects with type 2 diabetes (T2D), premeal consumption of protein and fat—either alone or in combination—has proved effective in decreasing or even normalizing postprandial hyperglycemia (Table 1). In 2006, Gentilcore et al. (22) demonstrated that 30 ml olive oil ingested 30 min before a carbohydrate meal was able to reduce and delay the postprandial glucose excursion in 6 diet-controlled T2D subjects. In 2009, the same group observed that a 55 g whey protein preload led to an even greater reduction in postprandial hyperglycemia in 8 diet-controlled T2D subjects (23). Thereafter, the ingestion of food rich in protein or fat before carbohydrate has been consistently associated with reduced postload glycemic excursions in T2D patients when compared with a carbohydrate-first meal pattern (18, 19, 27, 30, 31, 33–36, 39). On average, a ~40% reduction in glucose peak and a ~50–70% reduction in glucose excursion has been observed when protein and vegetables were consumed before carbohydrate, rather than mixed together or consumed after carbohydrate (33, 39). In our studies (18, 19), a small mixed protein and fat preload (50 g parmesan cheese and 50 g egg) was associated with a 30–50% reduction in glucose peak and overall excursion during an oral glucose tolerance test (OGTT) in well-controlled T2D patients. Similarly, Jakubowicz et al. (36) showed a ~30% reduction in postload glucose levels when 50 g whey protein were consumed before a high-glycemic index meal. Of note, the effect of macronutrient preloads on postprandial hyperglycemia in T2D appears comparable or even greater than that of current pharmacological therapy. In fact, Wu et al. (30) demonstrated that the glucose-lowering effect of a 25 g whey protein preload is similar to that of a dipeptidyl peptidase-4 (DPP-4) inhibitor (50 mg vildagliptin). Interestingly, combining the protein preload with vildagliptin was more effective for reducing postprandial glycemia compared with either treatment alone, thereby suggesting an additive effect. Further studies are needed to examine the interaction between nutrient preloads and oral hypoglycemic agents. In fact, preloading with saturated fat may lead to a deterioration in the glucose-lowering effect of DDP-4 inhibitors over time (48).

### Prediabetic Subjects

In individuals with impaired glucose tolerance (IGT), a mixed nutrient preload ingested 30 min before an OGTT was able to decrease postload glucose concentrations by 37% when compared with a water preload (18) (Table 1). In agreement with this finding, Shukla et al. (43) observed a similar reduction (−39%) in postprandial glycemia in IGT subjects who consumed protein and vegetables before carbohydrate, compared with the same foods consumed in the reverse order (i.e., carbohydrate before protein and vegetables). In 20 subjects with IGT and/or isolated 1-h glucose ≥160 mg/dl, a small (14 g) almond preload reduced postprandial glycemia by 15% (42). Interestingly, the effect was greater in individuals with higher 2-h glucose concentrations, suggesting an inverse correlation between the individual degree of glucose tolerance and the magnitude of the glucose-lowering effect achievable with nutrient preloads (42).

### Healthy Subjects

Nutrient preloads have been shown to reduce postprandial glucose concentrations even in subjects with normal glucose tolerance (NGT) (Table 1). Premeal consumption of either single amino acids (49, 50), whey protein (26, 44), a protein-enriched bar (27), dairy or soy milk (45), or margarine (21) before a carbohydrate-rich meal decreased postprandial glycemia in a dose-dependent manner in NGT subjects. Consistently, a mixed protein and fat preload reduced plasma glucose excursions after an OGTT by 32% in healthy young adults (18). Furthermore, the ingestion of either meat, fish, or vegetables before rice was able to decrease the postmeal glucose peak by ~50% and to delay it by 30–60 min when compared with eating the same food in the reverse order (i.e., rice first) (31, 46).

### Type 1 Diabetes

Despite recent improvements in insulin therapy, a tight control of postprandial hyperglycemia remains difficult to achieve in type 1 diabetes, and is frequently associated with an increased risk of insulin-induced hypoglycemia. In this setting, a recent study by Faber et al. (47) has shown that protein and fat consumed 15 min prior to carbohydrates reduced by ~10% mean glucose levels in 20 type 1 diabetic children and adolescents. Remarkably, the nutrient preload was not associated with an increased risk of hypoglycemic episodes (47).

### Gestational Diabetes

Glucose intolerance in pregnancy increases the risk of complications during delivery and the incidence of metabolic diseases later in life. In women with gestational diabetes (29), a treatment with low-carbohydrate preloads resulted in a significant reduction in both fasting and postprandial plasma glucose when compared with a dietary strategy implementing high-carbohydrate preloads. While low-carbohydrate preloads show promise, further studies are needed to determine efficacy and superiority of this approach.

## Long-Term Efficacy, Safety, and Feasibility

Despite numerous experimental studies demonstrated the acute beneficial effect of protein and fat consumption before carbohydrate on postprandial glycemia, only a few studies evaluated the long-term efficacy, feasibility, and safety of preload-based dietary strategies. In T2D subjects, a 25 g whey protein preload consumed 30 min before each meal for 4 weeks showed a sustained effect on postprandial glucose, with a nearly significant reduction in fructosamine levels (*p* = 0.06) (37). Furthermore, a 12-week intervention with mixed nutrient preloads was associated with decreased postprandial glucose and glycated hemoglobin levels in T2D subjects (28, 40). Finally, a recent study found a reduction in both fasting and postprandial plasma glucose in women with gestational diabetes consuming low-carbohydrate preloads for ~9 ± 1 weeks (29).

Dietary strategies that require nutritional supplements (either food or artificial formulas) might be expensive and poorly accepted. Moreover, although previous studies did not show weight gain after chronic preload consumption (28, 37) possibly due to the compensatory satiating effect of protein (26, 28, 51–55), adding nutrient preloads to each meal may increase the total daily caloric intake, leading to an increase in body weight and diet-related metabolic alterations (56, 57).

To limit the risk of weight gain and to increase the feasibility and cost-effectiveness of dietary interventions exploiting the same glucose-lowering effects of nutrient preloads, other strategies have been proposed. Low-calorie fiber-rich preloads (e.g., guar gum, vegetables), alone or in combination with protein, have been shown to improve glucose tolerance in both healthy and diabetic subjects with negligible effects on body weight (40, 41, 58–61). Furthermore, our group (20, 38) and others (31–33, 39, 43, 46, 60) have recently proposed a nutritional approach that simply consists in manipulating the sequence of macronutrient ingestion during each meal. In a proof-of-concept study, postprandial glucose control significantly improved in T2D subjects instructed to consume protein- and fat-rich food before carbohydrate-rich food for 8 weeks under free-living conditions, with no differences in body weight, serum lipid profile and other metabolic markers (38). These data support carbohydrate-last meal patterns as an effective and safe behavioral strategy to reduce postprandial glucose excursions.

## Physiological Mechanisms

### Gastric Emptying

The effect of non-carbohydrate nutrients on glucose tolerance is largely dependent on their ability to delay gastric emptying (18, 19, 22, 23, 31, 37, 45). Gastric emptying modulates the rate of oral glucose delivery and absorption in the small intestine, and it can account for about one third of the variance in the early glucose excursion during an OGTT (62–65). Fat is the most potent macronutrient in slowing gastric emptying (21, 62, 66–68). In 1989, Cunningham and Read (21) showed that the effect of fat on gastric emptying is greater when fat is consumed prior to carbohydrate rather than mixed with it, suggesting that this effect is dependent on the digestion of fat to fatty acids (22, 69, 70). In 2009, a protein preload was also found to be effective in slowing gastric emptying (23), as later confirmed by other groups (44). The effect of protein preloads on gastric emptying appears to be smaller compared to fat (23, 44, 71) and substantially unchanged after a 4-week consumption (37). Fat and protein may exhibit an additive effect on gastric emptying. In fact, a mixed protein and fat preload can markedly reduce oral glucose absorption across different classes of glucose tolerance (from −16% in NGT to−42% in T2D) (18) (Figure 1C). Consistently, Kuwata et al. (31) observed that both meat and fish consumed before rice are able to delay gastric emptying, particularly in T2D (31).

### Insulin Secretion and β Cell Function

The effect of nutrient preloads on postprandial glycemia largely depends on their insulinotropic action (18, 22, 23, 31, 33, 36, 50, 72). Among non-carbohydrate macronutrients, protein is the most effective in enhancing glucose-stimulated insulin secretion. Ma et al. (23) showed that a 55 g whey protein preload increases glucose-stimulated insulin release by 2- to 3-fold in T2D, and these results have been confirmed in both non-diabetic and T2D subjects (27, 36, 71). The insulinotropic effect of protein is dose-dependent (26) and likely mediated by both direct and incretin-mediated interactions of protein and amino acids with β cells (72–76). Although fat can enhance glucose-stimulated insulin secretion through direct and receptor-depended mechanisms (76–82), whether preloading with fat alone can affect insulin secretion is unclear. Indeed, the marked reduction in glucose responses due to the delay in gastric emptying after fat consumption usually leads to lower—rather than higher—absolute insulin levels in the early postprandial phase (21, 22). However, the insulin peak following a fat preload seems only delayed, but not reduced, even in the context of lower glucose levels (22). This observation suggests a positive—though small—effect of fat preloads on β cell function.

The effect of protein and fat on insulin secretion may be enforced by a synergistic interaction between the two classes of nutrients (18, 19, 83, 84). Our group showed that a mixed protein and fat preload increased plasma insulin levels during the first hour of an OGTT across the whole spectrum of glucose tolerance, despite lower glucose concentrations (18). The mixed preload increased the β cell responsiveness to plasma glucose (β cell glucose sensitivity) by 20% in NGT and prediabetic subjects, and almost doubled it in T2D subjects (Figure 1D). The greater enhancement of glucose-induced insulin secretion in T2D may be explained by a higher gradient of plasma amino acids after protein digestion and absorption in those subjects compared with healthy individuals (72, 85). Some studies with mixed preloads have reported different results (31, 39, 45, 46), likely due to a less rigorous estimation of β cell function (i.e., insulin and C-peptide levels were not adjusted for glucose concentrations) or to a stronger inhibition of gastric emptying by different preloads tested, which would minimize their impact on glucose-stimulated insulin secretion.

### Insulin Clearance and Insulin Sensitivity

Besides a direct stimulation of insulin release by pancreatic β cells, macronutrient preloads may increase insulin bioavailability by reducing insulin degradation (“insulin clearance”), which mostly occurs in the liver. In fact, we reported an average ~10% reduction in insulin clearance during a 2-h OGTT after a mixed nutrient preload, without significant differences between NGT, IGT and T2D subjects (18) (Figure 1E). A subsequent experiment showed a 52% increase in plasma insulin levels in T2D subjects during a 5-h OGTT, which was due to the combination of a 28% lower insulin clearance and a 22% higher insulin secretion after the nutrient preload (19).

Nutrient preloads may also impact on postprandial glucose homeostasis by affecting peripheral and hepatic insulin action (“insulin sensitivity”). However, no evidence so far has shown a significant influence of nutrient preloads on insulin sensitivity (18, 19).

### Incretin Hormones

Macronutrient preloads may exert their glucose-lowering effects by stimulating the release of gut hormones, such as the glucagon like peptide-1 (GLP-1) and the glucose-dependent insulinotropic polypeptide (GIP) (23, 44, 71, 86–91). GLP-1 and GIP are usually referred to as “incretin hormones” to underscore their stimulatory effect on pancreatic β cells, which is glucose-dependent, dose-dependent, and—only for GLP-1—largely preserved in T2D (86, 92). Furthermore, incretin hormones exhibit pleiotropic actions that include the inhibition of gastric emptying and appetite [by GLP-1 (86, 92)] and the reduction of hepatic insulin clearance [by GIP (90, 91)]. Preloading with either protein or fat alone enhanced GLP-1 concentrations in both T2D and healthy subjects, while only protein increased GIP levels in T2D (22, 23, 27, 36, 44, 71). When protein and fat were consumed together as a mixed preload, we observed an almost doubled GIP response, alongside with a modest but significant increase in plasma GLP-1 (18). These effects were comparable in individuals with different glucose tolerance, with a tendency to be more pronounced in IGT and T2D subjects (18, 19). Similarly, consuming meat or fish before carbohydrate resulted in higher GLP-1 and GIP concentrations in both T2D and healthy individuals, and these effects were greater in T2D (31).

### Additional Mechanisms

Several additional mechanisms have been proposed to explain the effect of non-carbohydrate nutrients on postprandial glucose control. Along with GLP-1 and GIP, protein (23, 44) and fat (93, 94) can stimulate the release of other gut hormones, such as cholecystokinin (CCK) and peptide YY (PYY), which inhibit gastric emptying and appetite (95–98) and stimulate insulin secretion (99–101).

The sight, smell and taste of nutrients may trigger neural signals leading to anticipatory insulin release, which may partly explain the insulinotropic effect of nutrient preloads (102). However, the contribution of the so-called “cephalic phase” of insulin secretion on glucose tolerance is little (~1% of postprandial insulin release), transient (8–10 min from sensory stimulation) (102), and not supported by experimental evidence (18).

Furthermore, it should be noticed that the glucose-lowering effect of nutrient preloads occurs despite an increase in plasma glucagon levels (18, 19, 31, 71), which is expected to worsen glucose tolerance by promoting gluconeogenesis and glycogenolysis. However, endogenous glucose production was not affected by premeal consumption of protein and fat (18, 19), and the relevance of increased glucagon concentrations in this setting remains controversial.

Finally, the reduction of appetite and calorie intake following protein consumption, which is possibly mediated by the stimulation of GLP1 secretion, might contribute to weight loss after long-term consumption of protein preloads (51–55).

## Conclusive Remarks and Future Perspectives

The experimental evidence discussed above indicates that premeal consumption of protein and fat can markedly reduce postprandial glycemia across the whole spectrum of glucose tolerance. The mechanisms underlying this effect include a delay in gastric emptying as well as an enhancement of glucose-stimulated insulin release and a decrease in hepatic insulin clearance, resulting, respectively, in slower glucose absorption and hyperinsulinemia (22, 23, 72). From the clinical perspective, the glucose-lowering effect of nutrient preloads is comparable in magnitude to that of current antihyperglycemic drugs (30), is proportionally greater in T2D than prediabetic and non-diabetic subjects (20), and appears to be sustained over time (37, 38). Preload-based dietary strategies can be useful in the management of T2D, either alone or in combination with pharmacological treatments, due to their additive effects (30). Furthermore, preload-based diets are of particular interest in clinical settings in which available pharmacological options are limited, including type 1 diabetes (47) and gestational diabetes (29), or not cost-effective, such as in the large number of individuals at high risk to develop T2D (18, 43). Remarkably, the same improvement in postprandial glycemia after nutrient preload consumption appears to be achievable by optimal timing of carbohydrate ingestion during a meal (i.e., carbohydrate-last meal pattern) (20, 31–33, 38, 39, 43, 46). This promising approach would avoid additional energy intake when compared with nutrient preloads, thereby minimizing the risk of body weight gain and diet-related metabolic alterations. Further refinement is required to determine the optimum timing and quantity of macronutrient consumption during a meal, as well as to standardize nutritional recommendations for targeting postprandial glycemia in different clinical settings. Larger studies are also needed to confirm the encouraging preliminary data on long-term efficacy, feasibility, and safety of these dietary approaches.

In summary, consistent experimental evidence suggests that preload-based nutritional strategies may offer a novel simple, effective, safe, and inexpensive therapeutic approach for the prevention and management of postprandial hyperglycemia and T2D.

## Author Contributions

LN and AM: data collection and analysis, interpretation of results, and manuscript writing; DT: funding, study design, data collection and analysis, interpretation of results, manuscript writing, and final editing. All authors read and approved the final submitted version of the manuscript.

### Conflict of Interest Statement

The authors declare that the research was conducted in the absence of any commercial or financial relationships that could be construed as a potential conflict of interest.
